# Improvement of Saccharification and Delignification Efficiency of *Trichoderma reesei* Rut-C30 by Genetic Bioengineering

**DOI:** 10.3390/microorganisms8020159

**Published:** 2020-01-23

**Authors:** Raja Mohan Gopalakrishnan, Tamilvendan Manavalan, Janani Ramesh, Kalaichelvan Puthupalayam Thangavelu, Klaus Heese

**Affiliations:** 1 Centre for Advanced Studies in Botany, University of Madras, Guindy Campus, Chennai, Tamil Nadu 600 025, India; rgrajamohan@yahoo.com (R.M.G.); tamilvendan@gmail.com (T.M.); 2 Department of Medical Biochemistry, Dr ALM Postgraduate Institute of Biomedical Sciences, University of Madras, Chennai, Tamil Nadu 600 113, India; rjjananiramesh95@gmail.com; 3 Graduate School of Biomedical Science and Engineering, Hanyang University, 222 Wangsimni-ro, Seongdong-gu, Seoul 133-791, Korea

**Keywords:** bioethanol, biomass degradation, delignification, *Ganoderma lucidum*, scanning electron microscope, *Trichoderma reesei*, versatile peroxidase

## Abstract

*Trichoderma reesei* produces various saccharification enzymes required for biomass degradation. However, the lack of an effective lignin-degrading enzyme system reduces the species’ efficiency in producing fermentable sugars and increases the pre-treatment costs for biofuel production. In this study, we heterologously expressed the *Ganoderma lucidum* RMK1 versatile peroxidase gene (*vp1*) in the Rut-C30 strain of *T. reesei*. The expression of purified 6×His-tag–containing recombinant *G. lucidum*-derived protein (rVP1) was confirmed through western blot, which exhibited a single band with a relative molecular weight of 39 kDa. In saccharification and delignification studies using rice straw, the transformant (tVP7, *T. reesei* Rut-C30 expressing *G. lucidum*-derived rVP1) showed significant improvement in the yield of total reducing sugar and delignification, compared with that of the parent *T. reesei* Rut-C30 strain. Scanning electron microscopy (SEM) of tVP7-treated paddy straw showed extensive degradation of several layers of its surface compared with the parent strain due to the presence of *G. lucidum*-derived rVP1. Our results suggest that the expression of ligninolytic enzymes in cellulase hyperproducing systems helps to integrate the pre-treatment and saccharification steps that may ultimately reduce the costs of bioethanol production.

## 1. Introduction

Lignocellulosic materials such as agricultural waste and crop residue, including rice straw, corn straw, cotton seed hair, cassava stem, and wheat straw, among others, have become feedstock for second-generation bioethanol production [1]. Extracting the maximum benefits from bioethanol production will require maximal utilization of lignocellulose materials [2], which consist of lignin and carbohydrate molecules such as cellulose, hemicellulose, pectin, etc. [3]. The presence of lignin hinders digestive enzymes’ access to cellulose and hemicellulose, which upon digestion serve as substrates for bioethanol production. The degradation of lignin is therefore essential for gaining access to the full contents of carbohydrates in efficient bioethanol production [4,5]. Generally, bioethanol production involves the preparation of feedstock (pre-treatment), digestion of cellulose and hemicelluloses into fermentable sugars (saccharification), and the conversion of fermentable sugars to bioethanol (fermentation) [6,7]. The pre-treatment step, which determines the quantity of fermentable sugars, and thus the quantity of bioethanol produced, improves the efficiency of lignocelluloses hydrolysis by separating cellulose from hemicellulose and lignin compaction [8,9]. Among the available methods, the physical methods are energy-consuming and the chemical methods are environmentally toxic, leaving biological treatment as the most viable pre-treatment option [9,10]. Integration of pre-treatment and saccharification using biological treatments is the preferred contemporary choice.

White-rot fungi represent more than 90% of wood-rot fungi and are the most efficient organisms in degrading lignin. Notable members of the group include *Ganoderma* spp., *Lentinula edodes*, *Phlebia radiata*, and *Pleurotus* spp. [11]. Lignin degradation by white-rot fungi is due to the production of various classes of lignin-modifying enzymes such as laccase (Lac), manganese peroxidase (MnP), lignin peroxidase (LiP), and versatile peroxidase (VP) [12,13,14]. High-redox-potential (0.8–1.2 V) enzymes, such as peroxidases, are more suitable for lignin degradation in terms of efficiency [15]. In this regard, VP is unique due to its high redox potential on the one hand and its substrate diversity on the other. Unlike MnP or LiP, it can oxidize both low- and high-redox compounds without an external mediator [16,17]. These advantages have drawn attention to delignification applications using VP [18,19].

*Trichoderma reesei*, the sexual anamorph of *Hypocrea jecorina*, is an industrially noteworthy soft-rot fungus known for its hyperproduction of cellulases and hemicellulases, which makes it an effective tool for biomass conversion industries [20]. It produces nearly nine different endoglucanases (EG) and two different cellobiohydrolases (CBH), all extracellularly. The cellobiohydrolases constitute nearly 85% of secreted proteins in *T. reesei* under cellulase-inducing conditions [21,22]. However, although *T. reesei* is capable of degrading crystalline cellulose, it is inefficient in degrading lignin [23]. The introduction of high-redox-potential enzymes, especially VP, should help the strain finding potential applications in biofuel industries. A successful attempt has been made to heterologously express a *P. radiata* laccase gene in *T. reesei*, which produced 20 mg/L of active laccase in fermentation at a small scale [24]. Another study yielded 28.6-fold higher laccase production by expressing a laccase gene from *Pleurotus ostreatus* in the *T. reesei* strain Tu6 [25]. Recently, a thermotolerant laccase from *Pycnoporus sanguineus* was expressed in *T. reesei* with a yield of 17.7 U/mL [26]. These studies on the expression of ligninolytic enzymes in *T. reesei* suggest that *T. reesei* can serve as a compatible host for effective ligninolytic enzymes production. Satisfactory protein secretion and post-translational modifications of *T. reesei* would make it a useful expression host, apart from the effortless downstream processing it offers [27]. In this investigation, we made an attempt to apply the engineered Pcbh1 promoter to heterologously express the RMK1 versatile peroxidase (*vp1*) gene from *Ganoderma lucidum,* an organism that houses different thermostable ligninolytic enzymes [28], into the *T. reesei* Rut-C30 strain to improve the biomass hydrolysis efficiency of the host organism. Upon generation of the transformant tVP7 (*T. reesei* Rut-C30 expressing *G. lucidum*-derived protein—rVP1) and rVP1 expression, we characterized purified *G. lucidum*-derived rVP1 and analyzed its delignification and saccharification efficiency using the transformant tVP7 while comparing it with its parent strain *T. reesei* Rut-C30.

## 2. Materials and Methods

### 2.1. Microbial Strains and Growth Conditions

*Ganoderma lucidum* RMK1 (accession number MH553170), isolated from Kumarakom Bird Sanctuary (9.6274° N, 76.4286° E) at Kumarakom, Kerala, India, was used as a source for the *vp1* gene (cDNA). *Trichoderma reesei* Rut-C30 (ATCC 56765) was used as the heterologous host for expression of the *G. lucidum*-derived *vp1* gene (JGI; Protein ID: 116056). Potato dextrose agar (PDA) was used to maintain fungal cultures throughout the study. *Escherichia coli* Top10-competent cells (Thermo Fisher Scientific, Waltham, MA, USA) cultivated in Luria-Bertani (LB) broth (HiMedia Laboratories Pvt. Ltd., Mumbai, Maharashtra, India) at 37 °C and 200 rpm overnight using 100 mg/mL kanamycin as a selection marker were used to propagate plasmids. *Agrobacterium tumefaciens* AGL1 [29] was used as a T-DNA donor for the transformation of the *vp1* gene into *T. reesei* Rut-C30 and was grown in LB medium at 28 °C under shaking conditions (200 rpm) for 36 h with hygromycin (100 mg/mL) as a selection marker. Two-day-old cultures of *G. lucidum* strain RMK1 and *T. reesei* strain Rut-C30 cultivated in potato dextrose broth (PDB) were used for genomic DNA isolation following the cetyltrimethylammonium bromide (CTAB) method [30]. Further details about the materials used in these experimental procedures are provided in the Supplementary Materials.

### 2.2. Construction of Cloning Vector

The binary vector pCambia 1300 (CAMBIA, Canberra, Australia) was used as the backbone for the construction of the plasmid vector and as the recipient for the construction of the T-DNA binary vector. The *hph* gene that codes for hygromycin B phosphotransferase was first introduced into the vector under the control of the *Aspergillus nidulans trpC* promoter and terminator to create the pCXH vector [31]. The *cbh1* promoter Pcbh1 and the *xyn2* gene were amplified using the primer pairs of Pcbh1F, Pcbh1R, Xyn2F and Xyn2R (Table S1), respectively, using the *T. reesei* Rut-C30 genomic DNA as a template. The coding region of the *vp1* gene was amplified using *G. lucidum* RMK1 genomic DNA as a template with the respective forward and reverse primers listed in Table S1. The amplified sequences were separated by agarose gel electrophoresis using a horizontal submarine electrophoresis apparatus (Medox, Chennai, Tamil Nadu, India). The separated sequences of interest were observed under an ultraviolet transilluminator and immediately excised using a sterile blade and purified using Axygen’s gel extraction kit. The purified promoter (*cbh1*), genes (*xyn2 and vp1*) with a 6×His-tag (at the C-terminus of rVP1), and terminator segments were fused to the linearized pCXH vector at the predetermined cloning site (Xba1) using the In-Fusion HD cloning kit (Bio-Rad). The resulting constructed vector (pCXH-VP1) (Figure S1) was propagated in *E. coli* Top10 cells and the purified plasmid was transformed into *A. tumefaciens* AGL1.

### 2.3. Transformation of vp1 into T. reesei Rut-C30

Transfer of the *vp1*-containing vector to *T. reesei* Rut-C30 was carried out using *A. tumefaciens*- mediated transformation as described previously [31]. Transformed *A. tumefaciens* AGL1 (carrying the plasmid vector pCXH-VP1) was grown for 36 h at 28 °C in LB medium containing kanamycin and carbenicillin. The culture was then diluted in an induction medium (IM) containing 10 µL of 200 μM acetosyringone (AS) and 400 µL of 1 M 2-(N-morpholino) ethanesulphonic acid (MES) to attain an optical density (OD_660_) of 0.15. The *A. tumefaciens* AGL1 culture was again grown at 28 °C for 6 h or until the culture reached an OD_660_ of 0.6 in IM medium (10 mL, with added AS and MES). In another step, *T. reesei* Rut-C30 was grown for 7 days and the spores were extracted using 1 mL of a spore-extraction solution containing Tween 80 (0.05%) and NaCl (0.85%) and the spore concentration was adjusted to 10^6^/mL. Equal volumes of *A. tumefaciens* AGL1 culture and *T. reesei* Rut-C30 conidia were smeared on an IM plate containing 200 μM AS (50 µL) and MES (2 mL) and co-cultured for 2 days at 26 °C in the dark. The co-cultured plate was supplemented with 1 mL of saline solution (gently smeared with a sterile glass rod). The smeared co-culture paste was then spread onto PDA medium containing 2 mL of 10% (*v/v*) Triton-X 100, hygromycin (0.37 µM), and cefotaxime (10 µM), and incubated at 37 °C. Well-distinguished round-shaped colonies appeared after 5 to 7 days of incubation and the colonies were transferred onto 24-well PDA plates (supplemented with hygromycin B [0.37 µM] and cefotaxime [10 µM] antibiotics at pre-determined concentrations). The integration of the *vp1* gene into the genomic DNA of *T. reesei* Rut-C30 was verified by polymerase chain reaction analysis using the forward and reverse primers of the *vp1* gene.

### 2.4. Enzyme Activity of rVP1 and Protein Assay

Experimental details of production and purification of rVP1 using transformed *T. reesei* Rut-C30 are provided in the Supplementary Materials. Briefly, the crude culture filtrate from 1-week old *T. reesei* Rut-C30 (parent and transformant tVP7) was centrifuged at 12,000× *g* for 15 min at 4 °C for the enzyme assay and protein estimation. Protein concentration in both the culture filtrates was estimated using a commercial kit (Bio-Rad DC Protein Assay Kit; Bio-Rad Laboratories, Inc., Hercules, CA, USA) in which bovine serum albumin (BSA) served as a protein standard. rVP1 was quantified by measuring the oxidation of phenol red (A_610_ ε = 22.0 mM^−1^cm^−1^) at Mn^2+^-independent conditions. The 1.5 mL reaction mixture contained 0.001% (*w/v*) phenol red, 2.5 mM lactate, 0.01% (*w/v*) BSA in a 20 mM sodium acetate buffer (pH 4.5). The oxidation reaction was stopped by the addition of NaOH at a final concentration of 8 mM. The measurements were taken in the presence and absence of H_2_O_2_ for measuring the peroxidase-specific activity. Boiled samples of culture filtrates (otherwise the same conditions) were used as blanks. One unit of enzyme activity was equivalent to the amount of enzyme that catalyzed the production of 1 μM of oxidation product per mL of culture filtrate. Additional methodological details for electrophoresis and western blot analyses are provided in the Supplementary Materials (Materials and Methods section).

### 2.5. Delignification and Saccharification of Paddy Straw by Transformants and Parent T. reesei Rut-C30

Fresh paddy straw was collected from a local paddy field (Chennai, Tamil Nadu, India). The composition of cellulose, hemicelluloses, and lignin in the paddy straw was analyzed as described previously [32]. The experiment was carried out in triplicate. To compare the delignification efficiency of transformed *T. reesei* Rut-C30 strain tVP7, the two paddy straw samples (0.5 g/mL) were pre-treated [33] with acid (5% *w/v* H_2_SO_4_) and alkali (5% *w/v* NaOH), and a third sample of paddy straw was subjected to simultaneous pre-treatment and saccharification using the culture filtrate of transformed *T. reesei* Rut-C30 strain tVP7. For pre-treatment, 0.5 g of paddy straw sample was added with 10 mL of 5% (*w/v*) NaOH or H_2_SO_4_ solution and incubated at 50 °C for 2 h under shaking condition (150 rpm). Then, the pre-treated samples were centrifuged at 3000 *g* for 5 min. and washed thrice with water. Before the acid pre-treatment, the sample was autoclaved at 121 °C (15 psi) for 20 min. The pre-treated pellet was lyophilized and stored at −20 °C until used further. About 100 mg/mL of the lyophilized pre-treated biomass was suspended in acetate buffer (50 mM, pH 4.5) in a 2 mL Eppendorf tube for saccharification. To all three sets of preparations, 3 filter paper units (FPUs) of cellulase enzyme (from parent *T. reesei* Rut-C30) per gram of substrate were added. The saccharification/hydrolysis was carried out at 45 °C under shaking conditions at 150 rpm for 48 h. The amount of lignin in the pre-treated samples was quantified as described previously [32] to measure the delignification efficiency of different pre-treatments. Saccharification efficiency was estimated by quantifying the production of total reducing sugar (TRS) using the 3,5-dinitrosalicylic acid (DNS) method [34].

### 2.6. Scanning Electron Microscopy

Morphological and structural changes in paddy straw during pre-treatment were observed using SEM. The paddy straw pre-treated with supernatants of transformed and untransformed parent strains were compared with untreated paddy straw. The pre-treated paddy straw residue was washed with water and finally lyophilized for SEM analysis. The dried samples were coated with gold (Polaron Emitech SC7640 sputter coater, UK) and microscopic images were taken at 250X by a PhenomPro desktop SEM (Phenom-World, Eindhoven, The Netherlands) at a voltage of 10 kV.

### 2.7. Statistical Analysis

The data obtained in this study are presented as mean ± standard deviation. The differences between the groups were established using an unpaired Student’s *t*-test, while within-group comparisons were performed using the paired Student’s *t*-test.

## 3. Results

### 3.1. Construction and Transformation of Expression Vector Containing vp1 into T. reesei Rut-C30

The expression vector was successfully constructed and the final arrangement of the gene-vector construct (named pCXH-VP1) read like pCXH-Pcbh1-Xyn2-VP1-Ttrpc-pCXH in a circular clock-wise fashion (Figure S1). The transformation of the constructed vector into *T. reesei* Rut-C30 was confirmed through colony PCR and, out of 20 colonies tested, 2 colonies, tVP7 and tVP13, showed successful transformation. The transformation efficiency of *T. reesei* was calculated to be between 250 and 400 successful transformants per 1 × 10^7^ conidia. This confirmed the successful integration of the *vp1* gene into the chromosomal DNA of *T. reesei* Rut-C30.

### 3.2. Enzyme and Protein Production by Transformants and Parent Strain T. reesei Rut-C30

To express and purify heterologous rVP1 protein in *T. reesei* Rut-C30, a 6×His-tag was introduced to the C-terminus of the rVP1 protein. Both successful transformants (tVP7 and tVP13) were cultivated in fermentation medium along with the parent *T. reesei* Rut-C30 strain and all culture filtrates were harvested and analyzed for protein quantity and VP activity. The supernatant of transformant tVP7 showed higher enzyme activity with 437 ± 1.0 U/L of VP activity and produced 10.52 ± 0.01 mg/mL of extracellular protein. The transformant tVP13 produced comparatively less enzyme (384 ± 1.08 U/L) and protein (9.48 ± 0.02 mg/mL). By comparison, the parent *T. reesei* Rut-C30 produced 9.08 ± 0.03 mg/mL of protein and no VP (Table 1). The filter paper activity (FPA) of the two transformants (tVP7 and tVP13) and parent *T. reesei* Rut-C30 were 14.92 ± 0.32, 12.36 ± 0.25, and 13.61 ± 0.24 U/mL, respectively. This showed that the introduction of the *vp1* gene did not affect FPA significantly in tVP7 and tVP13 transformants (*p* = 0.154 and 0.134, *t*-test) compared with their parent *T. reesei* Rut-C30 strain.

**Figure 1:**
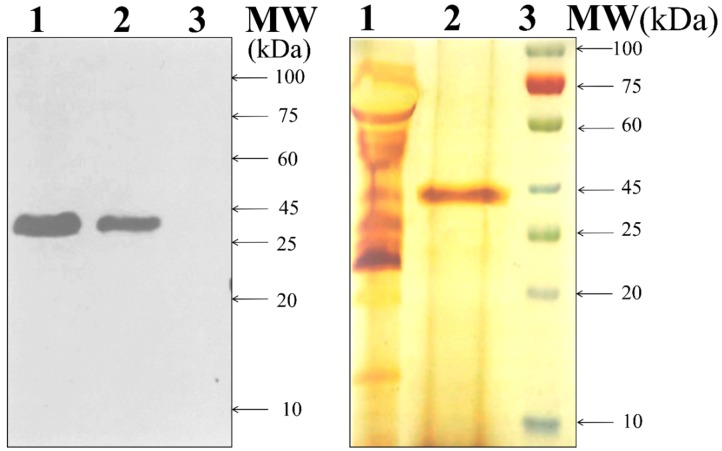
Molecular weight determination of rVP1. Left panel: Western blot analysis of extracellular recombinant *G. lucidum*-derived protein (rVP1) expressed using transformed *T. reesei* Rut-C30 strains tVP7 and tVP13. Lane 1, crude culture supernatant of transformant tVP7; lane 2, crude culture supernatant of transformant tVP13; lane 3, crude culture supernatant of the parent strain (*T. reesei* Rut-C30); molecular weight (MW) in kDa; right panel: molecular weight determination of rVP1 from transformed *T. reesei* Rut-C30 strain tVP7. Lane 1, crude culture supernatant from transformant tVP7; lane 2, purified rVP1 from transformant tVP7; lane 3, pre-stained protein marker (in kDa).

The successful expression of rVP1 was confirmed using western blot analysis by analyzing the extracellular proteins of the two positive transformants (tVP7and tVP13 strain) and comparing them with the parent *T. reesei* Rut-C30 strain. Western blot analysis showed a clear band at a molecular weight of 39 kDa in the tVP7 and tVP13 transformants carrying the *G. lucidum*-derived *vp1* gene, which is close to the theoretical value of ~37 kDa (w/o VP1 signal peptide) calculated from the deduced amino acid sequences for the *vp1* gene from *G. lucidum* RMK1 (Figure 1, left panel). The rVP1 enzyme was purified using single-step purification and Ni-NTA affinity column chromatography (Qiagen). The purified rVP1 exhibited 4.68-fold purity compared with the crude culture filtrate (Table 1). The purity of the final rVP1 preparation was analyzed by SDS-PAGE followed by silver nitrate staining [35], which confirmed the presence of a single polypeptide chain with a relative molecular mass of 39 kDa (Figure 1, right panel).

### 3.3. Delignification and Saccharification Efficiency of Transformed T. reesei Rut-C30

Cellulose was the most abundant component in the paddy straw with a proportion of 43.5% ± 1.2%, followed by hemicellulose with 23.2% ± 0.4% proportion. The holocellulose (cellulose and hemicellulose) constituted approximately 66.70% of the total dry weight of the paddy straw. The paddy straw was estimated to contain 16.1% lignin, for which the acid-soluble and acid-insoluble lignin content was 3.8% ± 0.1% and 12.3% ± 0.3%, respectively. The amount of ash was estimated to be 11.7% ± 0.4% of the total dry weight of the paddy straw. The balance of the proportion (to 100%) was assumed to be the percentage of silica present in the paddy straw.

**Figure 2:**
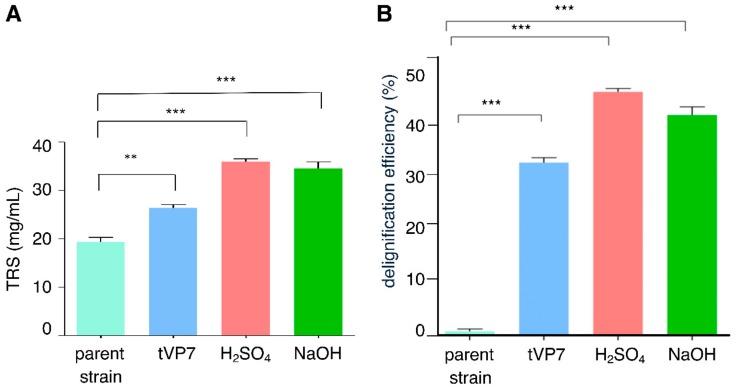
Saccharification and delignification efficiency of transformant tVP7. (**A**) Saccharification of paddy straw as total reducing sugar (TRS) after different pre-treatments. Values represent data obtained in triplicate assays. (**p < 0.01 and ***p < 0.001, compared with the parent strain *T. reesei* Rut-C30); (**B**) Delignification efficiency of different pre-treatments using paddy straw as lignocellulosic biomass. Values represent data obtained in triplicate assays. (***p < 0.001, compared with the parent strain *T. reesei* Rut-C30).

The ground paddy straw was initially pre-treated with NaOH and H_2_SO_4_, followed by simultaneous pre-treatment and saccharification with supernatants of cloned and parent *T. reesei* Rut-C30 culture filtrates. Among the four different tested conditions, paddy straw pre-treated with sulfuric acid exhibited maximum saccharification, with a maximum total reducing sugar of 35.84 mg/mL, followed by NaOH pre-treatment, which yielded 33.56 mg/mL of total reducing sugar. The transformed *T. reesei* Rut-C30 strain tVP7 and parent *T. reesei* Rut-C30 treated paddy straw produced 25.45 and 18.69 mg/mL of total reducing sugar, respectively (Figure 2A).

Lignin quantification after the different pre-treatments was carried out according to the method cited earlier [32]. The maximum delignification of 46.95% was achieved during dilute acid pre-treatment (5% *v/v* H_2_SO_4_) followed by alkaline pre-treatment (5% *w/v* NaOH), which achieved delignification of 43.72%. The transformed *T. reesei* Rut-C30 strain tVP7 achieved a delignification efficacy of 32.68% (Figure 2B).

### 3.4. Scanning Electron Microscopic Observation

**Figure 3:**
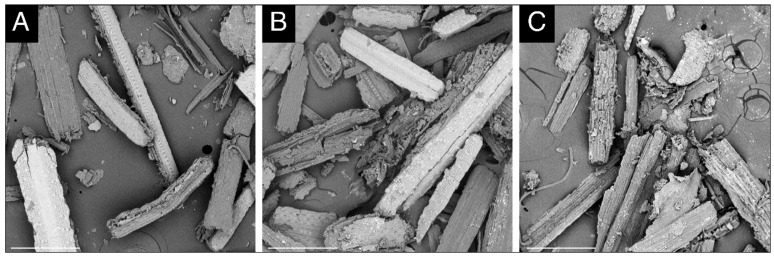
SEM analysis of paddy straw (representative pictures are shown): (**A**) untreated paddy straw, (**B**) paddy straw treated with culture filtrate of parent *T. reesei* Rut-C30, and (**C**) paddy straw treated with culture filtrate of transformed *T. reesei* Rut-C30 strain tVP7. Scale bars represent 300 µm.

The effect of crude culture filtrates of transformed *T. reesei* Rut-C30 strain tVP7 and parent *T. reesei* Rut-C30 with respect to the control (untreated) on the topology of paddy straw is shown in Figure 3A–C. A close look at untreated paddy straw (Figure 3A) reveals the presence of phytoliths (silica-storing bodies) on an intact epidermis. The treatment of paddy straw with the culture filtrate of the parent strain exhibited signs of saccharification, which is indicated by the rugged and irregular surface and the absence of phytoliths (Figure 3B). The crude extract of transformed *T. reesei* Rut-C30 (tVP7) exhibited severe distortion of several layers of paddy straw because of the saccharification and delignification due to the presence of rVP1 (Figure 3C).

## 4. Discussion

*Trichoderma reesei* is one of the most important filamentous fungi among model organisms for the production of recombinant protein and in plant biomass degradation [36]. After the discovery of transformation techniques [37,38], various proteins of fungal and non-fungal origin have been successfully expressed in *T. reesei* [39,40]; the production of fungal proteins, in particular, was more efficient than that of mammalian proteins. For instance, fungal proteins such as laccase, lipase, xylanase, and glucose oxidase were expressed using the *cbh1* promoter at levels of 100 mg/L and several grams per liter in shake-flask and fermentation cultures, respectively [26,41,42,43]. One of the most advantageous properties of *T. reesei* is that it does not hyperglycosylate proteins. *Trichoderma reesei* produces significantly less protease than *Aspergillus nidulans* [44], allowing the former to be used as a host for heterologous gene expression.

In the present study, we cloned the *vp1* gene from *G. lucidum* RMK1 and expressed rVP1 in *T. reesei* Rut-C30. Heterologous expression of the *vp1* gene was carried out under the control of the strong wild-type *cbh1* promoter as suggested by earlier reports [45]. The *cbh1* promoter encodes cellulase in *T. reesei* and is a default promoter for heterologous expression of proteins in *T. reesei* [46,47]. The use of the *cbh1* promoter was further supported by a recent study [48] that claimed successful over-expression of *Aspergillus oryzae* acetyl xylan esterase in *T. reesei* under the control of the *cbh1* promoter.

Lack of a generous cell wall facilitating DNA transfer makes filamentous fungi difficult to use in transformation experiments [49]. Owing to the complex processes involved in protoplast fusion, a research group found *A. tumefaciens* AGL1 to be efficient for the transformation of T-DNA to the host through a co-cultivation method and AS amendment [50]. Here, we applied the same method to transform the *vp1* gene from *A. tumefaciens* AGL1 to *T. reesei* Rut-C30 with an efficiency of 250 to 400 successful transformants per 1 × 10^7^ conidia.

We attempted to express *vp1* for the secretion of rVP1 using the native signal peptide, but the transformants failed to secrete the protein extracellularly. In most cases of heterologous protein expression, the poor native signal peptide in the gene of interest explained the host inefficiency in secreting the gene product, even after successful transformation [51]. A recent report suggested that the fusion of the *xyn2* gene to the gene of interest is a novel idea for the successful secretion of the gene product, especially for heterologous expression of genes using *T. reesei* as the host [48]. Accordingly, we fused the *T. reesei xyn2* gene with the *G. lucidum vp1* gene. The product rVP1 enzyme was secreted extracellularly upon post-translational cleavage from XYN2 during fermentation by the transformants tVP7 and tVP13, which are absent in the parent *T. reesei* Rut-C30 strain, as confirmed by western blot analysis. Our purified rVP1 enzyme measured 39 kDa in the purified gel, a size similar to the native VP1 and suggestive of proper processing in the host system. While other researchers have reported a VP larger than ours, a purified VP with a molecular mass of 38 kDa, which is closer in size to our purified enzyme, has also been reported [52].

Homologous and heterologous expression of VP has been studied in a variety of prokaryotic and eukaryotic hosts (Table 2) [19,46,52,53,54,55,56,57,58,59,60,61,62,63,64,65]. Expression of *Bjerkandera adusta* VP in prokaryotic *E. coli* BL21 (DE3) yielded 322 U/L of rVP [58]. However, expression of *Pleurotus eryngii* VP in eukaryotic *Aspergillus nidulans* and *A. niger* resulted in a yield of 466 U/L and 412 U/L active enzymes, respectively [59]. The heterologous expression of *Pleurotus eryngii* VP in *Phanerochaete chrysosporium* [63] produced 20 U/mL of enzyme, the largest quantity of VP by heterologous expression reported to date. However, no research has been carried out on the expression of a VP gene in *T. reesei*. In our investigation, we obtained a maximum rVP1 enzyme production of 437 U/L from our transformant tVP7. The use of the *cbh1* promoter increased the efficiency of rVP1 production in *T. reesei* Rut-C30.

Paddy straw, one of the most common forms of agricultural waste in Asia, provides an opportunity for sustainable bioethanol production due to its high potential for total reducing sugar (in terms of high cellulose content) and low lignin content [66]. Despite a lower content of lignin, pre-treatment is necessary for efficient saccharification of paddy straw and further fermentation to ethanol. For example, only 20% of the total reducing sugar can be extracted from native paddy straw by enzymatic hydrolysis without pre-treatment [67]. Pre-treatment can therefore significantly improve the saccharification yield of the paddy straw [68]. In our experiment, paddy straw saccharified with supernatant of parent *T. reesei* Rut-C30 yielded 18.69 mg/mL of reducing sugar. The transformed *T. reesei* Rut-C30 strain tVP7 improved the yield of reducing sugar to 25.45 mg/mL. An earlier study obtained 268.5 mg/g reducing sugar from 4% NaOH-pre-treated paddy straw on saccharification with *T. reesei* Rut-C30 with a saccharification rate of 54.3% [69]. In this study, we obtained 33.56 mg/mL of reducing sugar by pre-treating paddy straw with 5% NaOH (Figure 2A). A recent study reported a 4.5-times improvement in glucose yield (43.5 g/L) from paddy straw (9.5 mg/mL) treated with 0.5 M Na_2_CO_3_ at 180 °C for 120 min compared with untreated paddy straw [70].

We obtained the greatest amount of reducing sugar (approximately 35.84 mg/mL) in the acid pre-treatment and saccharification using the *T. reesei* Rut-C30 culture filtrate (Figure 2A). Consistent with our results, a previous study [71] obtained 359 mg of reducing sugar per gram of paddy straw at optimized conditions (pre-treatment: 0.5% sulfuric acid for 60 min at 121 °C in an autoclave; saccharification: 40 FPU/g enzymes, 17.50% substrate, at 50 °C for 72 h) that differed from ours. Another pilot-scale study that applied diluted acid pre-treatment to rice straw obtained 115.2 mg/mL of total reducing sugar at conditions of 0.35% (*w/v*) H_2_SO_4_ and 162 °C for 10 min [72]. Our observations were corroborated with SEM analysis of treated and untreated paddy straw. SEM observations of paddy straw treated with culture filtrates of transformed tVP7 and parent *T. reesei* Rut-C30 revealed severely distorted topology (Figure 3). The absence of such modifications in untreated and parent-treated paddy straws confirmed the efficiency of our transformed *T. reesei* Rut-C30 strain tVP7. Similar structures of phytoliths on an intact epidermis and rugged cell structures were reported with untreated and NaOH treated paddy straw surfaces, respectively, under SEM observation [73].

Our delignification study revealed the transformant tVP7 efficiently removed lignin, although not as effective as the acid and alkali pre-treatments. Other teams have reported higher levels of delignification, using acid and alkali methods [70,74]. However, the improved saccharification yield and delignification reported in other studies were only due to the application of higher temperatures during pre-treatment, and applying high temperatures may severely affect production costs. Although chemical pre-treatments produced more fermentable sugars than the cloned tVP7 and parent *T. reesei* Rut-C30 strains-derived supernatants (with paddy straw as a substrate), the fermentation efficiency of H_2_SO_4_ (80.17%) and NaOH (84.08%) treatments were relatively low (unpublished observation), likely due to the production of toxic by-products [75]. Pre-treatment of paddy straw increases the accessibility and surface area of cellulose by lignin modification or degradation, leading to enhanced saccharification efficiency. Incidentally, our transformed *T. reesei* Rut-C30 strain tVP7 hydrolysate achieved a maximum fermentation efficiency (93.85%), followed by its parent *T. reesei* Rut-C30 strain (87.99%) (unpublished observation), emphasizing the importance of the biological pre-treatment process to improve the efficiency of fermentation and ethanol production.

## 5. Conclusions

**Figure 4:**
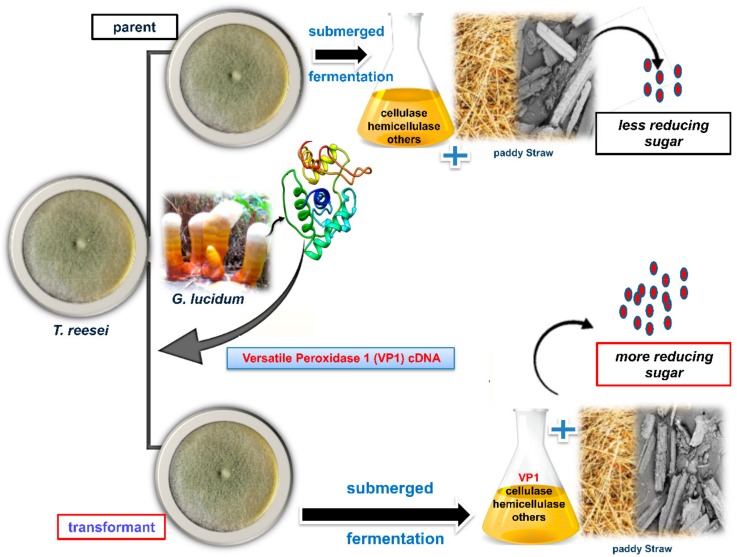
A schematic summary showing increased reducing sugar yield for improved bioethanol production using *T. reesei* through a genetic bioengineering approach.

In the current study, the VP1 encoding gene (*vp1*) was cloned from *G. lucidum* RMK1 and heterologously expressed in *T. reesei* Rut-C30 under the control of a strong and inducible *cbh1* promoter. The production of rVP1 by the transformed *T. reesei* Rut-C30 strain tVP7 improved the saccharification efficiency of *T. reesei* Rut-C30, which lacks efficient endogenous ligninolytic enzyme activity. These results make rVP1 a promising candidate for the efficient degradation of lignocellulosic biomasses (Figure 4). Such a genetic bioengineering approach could also provide an opportunity to heterologously express rVP1 in other industrial cellulase producing strains and reduce the costs of ligninolytic enzyme production for biofuel applications and lignin valorization.

## Figures and Tables

**Table 1:** Protein concentration and enzyme activity of parent *T. reesei* Rut-C30 and transformed *T. reesei* Rut-C30 strains tVP7 and tVP13.

Strain	Extracellular (mg/mL)	Protein VP Activity (U/L)	FPA (U/mL)	VP-Specific Activity (U/mg)
*T. reesei* Rut-C30	09.08 ± 0.03	ND	13.61 ± 0.24	ND
tVP7	10.52 ± 0.01	437 ± 1.0	14.92 ± 0.32	41.53 ± 1.02 ***
tVP13	09.48 ± 0.02	384 ± 1.08	12.36 ± 0.25	40.50 ± 1.04 ***

Values represent data obtained from triplicate assays. FPA, filter paper activity; ND, not detectable; *** *p* < 0.001, compared with the parent *T. reesei* Rut-C30 strain.

**Table 2:** Comparative details of versatile peroxidase (VP) cloned in different prokaryotic and eukaryotic expression hosts.

Source	Host	Vector	Promoter	Enzyme Yield	Reference
*Physisporinus vitreus*	*E. coli* BL21(DE3)	pET-28a(+)	default	18 mg/L	[19]
*Pleurotus eryngii*	*Emerciella nidulans*	-	*alcA*	165 U/L	[46]
*Pleurotus sapidus*	*Hansenula polymorpha* RB11	pFPMT121	default	1.80 ± 0.10 U/mg specific activity	[52]
*Pleurotus eryngii*	*Aspergillus nidulans*	palcA1	*alcA*	294.3 mg	[53]
*Pleurotus eryngii*	*E. coli* W3110	pFFLAG1	*tac*	-	[54]
*Pleurotus ostreatus*	*Pleurotus ostreatus*	pIpM2g	*sdi I*	1.5 mg/L	[55]
*Pleurotus ostreatus*	*Pleurotus ostreatus*	pIpMc	*sdi I*	370 mU/mL	[56]
*Pleurotus eryngii*	*E. coli* W3110	pFFLAG1	*tac*	5.5 mg/L	[57]
*Bjarkendra adjusta*	*E. coli* BL21(DE3)	pET-32b(+) and pET-19b(+)	default	322 U/L	[58]
*Pleurotus eryngii*	*Aspergillus nidulans*,	PAN7-1	*alcA*	466 U/L (*A. nidulans*);	[59]
	*Aspergillus niger*			412 U/L (*A. niger*)	
*Pleurotus eryngii*	*E. coli* W3110	pFLAG-VPL2	default	-	[60]
*Pleurotus eryngii*	*E. coli* BL21(DE3)	pET-28a(+) and pET-32a(+)	default	12.5 mg/L	[61]
*Pleurotus ostreatus*	*Pleurotus ostreatus*	pTM1	β-tubulin promoter	96 mU/mg substrate	[62]
*Pleurotus eryngii*	*Phanerochaete chrysosporium*	Ppchph	*gpd*	20 U/mL	[63]
*Moniliophthora roreri*	*Pichia pastoris*	pPICZaA	*aox1*	295 mg/L	[64]
*Irpex consors*	*E. coli* Top 10	-	-	> 250 U/L	[65]
*Ganoderma lucidum*	*T. reesei* Rut-C30	pCambia-1300	*cbh1*	437 U/L	present study

-: information not provided.

## Supplementary Materials

Supplementary Materials can be found at https://www.mdpi.com/2076-2607/8/2/159/s1.
