# Supplementary material for: Improvement of Saccharification and Delignification Efficiency of Trichoderma reesei Rut-C30 by Genetic Bioengineering
Source: Microorganisms. 2020 Jan 23;8(2):159. doi: 10.3390/microorganisms8020159 (PMC7074786; doi:10.3390/microorganisms8020159)
Supplement: Supplementary file 1 [file microorganisms-08-00159-s001.pdf]

microorganisms

Improvement of Saccharification and Delignification Efficiency of *Trichoderma reesei* Rut-C30 by Genetic Bioengineering

Supplementary Materials and Methods

Materials

All the chemicals used herein were of analytical grade and were purchased from Sigma Aldrich (Bengaluru, Karnataka, India). The MAGSPIN-34 MAGUniversal blU prestained protein ladder was acquired from APS LABS (Pune, Maharashtra, India). Cloning experiments were carried out using the In-Fusion HD cloning kit obtained from Takara (Mountain View, CA, USA). Plasmid DNA purification and DNA gel extraction kits were purchased from Axygen Biosciences (Union City, CA, USA). The Xba1 restriction enzyme was procured from Thermo Fisher Scientific (Waltham, MA, USA). Protein concentrations were estimated using a commercial kit (Bio-Rad DC Protein Assay Kit, Bio-Rad Laboratories, Inc., Hercules, CA, USA). Anti 6×His-tag antibody and horseradish peroxidase (HRP)-conjugated AffiniPure goat anti-mouse immunoglobin G (IgG) were obtained from Proteintech Group (Rosemont, Illinois, USA). To detect HRP activity in western blots, Pierce enhanced chemiluminescence (ECL) western blotting substrate was purchased from Thermo Fisher Scientific India Pvt. Ltd. (Powai, Mumbai, India).

rVP1 Production by Fermentation of Transformed T. reesei Rut-C30 Strain tVP7

Conidial suspension of transformed *T. reesei* Rut-C30 tVP7 was prepared by dispersing 2 mL of spore-extraction solution on potato dextrose agar (PDA) containing 1-week old actively growing mycelium. The spore solution was harvested and filtered using spore filter vials to remove the mycelium in the collection. Approximately 2.5 mL of the conidial suspension of transformed *T. reesei* Rut-C30 tVP7 was inoculated into 10 mL of sterile potato dextrose broth (PDB) in a 50 mL Erlenmeyer flask. The inoculated medium was incubated at 28 °C for 2 days under shaking conditions (200 rpm). The actively growing two-day-old culture was inoculated into fermentation medium at an inoculum ratio of 10% v/v. The culture was incubated at 28 °C for 7 days under shaking conditions at 200 rpm. The fermentation medium had the following composition: KH_2PO_4 (0.4%), $(NH_4)_2SO_4$ (0.28%), $MgSO_4{\cdot}7H_2O$ (0.06%), $CaCl_2$ (0.05%), urea (0.06%), tryptone (0.3%), Tween 80 (0.1%), $CaCO_3$ (0.5%), $FeSO_4{\cdot}7H_2O$ (0.001%), $MnSO_4{\cdot}H_2O$ (0.00032%), $ZnSO_4{\cdot}7H_2O$ (0.00028%), $CoCl_2$ (0.0004%), wheat bran (2%), Avicel (1%), and teakwood lignin (0.2%). After 7 days of fermentation, the rVP1-containing culture supernatant was harvested by centrifugation at 12,000 *g* for 5 min at 4 °C.

Purification of rVP1

The rVP1 enzyme containing a 6×His-tag was purified from a crude culture filtrate of transformed *T. reesei* Rut-C30 strain tVP7 (*vp1* gene transformant tVP7 [rVP1 containing a 6×His-tag]) at 4 °C using single-step nickel–nitrilotriacetic acid (Ni-NTA) affinity column chromatography following the manufacturer's protocol (Qiagen India Pvt. Ltd., Karol Bagh, New Delhi, India). Initially, the ethanol solution in the column, used for storage of stationary phase material, was emptied and the column was washed sufficiently with double-distilled water followed by a wash with phosphate-buffered saline (PBS). The culture filtrate was added to the column and incubated for 2 h. The culture filtrate was then allowed to run through the column, collected, and stored separately. Unbound proteins were washed away with PBS and the loosely bound proteins were washed consecutively with 20 mM, 40 mM, and 60 mM imidazole, respectively. Strongly bound 6×His-tag–containing rVP1 was eluted with 2 mL of 150 mM imidazole. Thereafter, the column was

restored by washing sequentially with 500 mM imidazole, PBS buffer, double-distilled water, and finally 20% ethanol for storage at 4 °C. The purity of rVP1 was analyzed by sodium dodecyl sulfate polyacrylamide gel electrophoresis (SDS-PAGE). The fractions exhibiting enzymatic activity and exhibiting a single band in SDS-PAGE were pooled and dialyzed twice against 10 mM sodium acetate (pH 5.5) and finally concentrated using Amicon Ultra-15 centrifugal filters (cut-off 3 kDa) from Merck (Mumbai, Maharashtra, India).

Electrophoresis and Western Blot Detection of rVP1

Extracellular culture supernatants were collected from a 1-week old culture of *T. reesei* Rut-C30 and the transformed *T. reesei* Rut-C30 strain tVP7 (*vp1* gene transformant tVP7 [rVP1 containing a 6×His-tag]) after centrifugation at 12,000 *g* for five minutes. SDS-PAGE (12%) was then carried out to separate the proteins for 1 h and 30 min at 100 V. After the SDS-PAGE was completed, half of the gel was subjected to silver nitrate staining [1] and the other half was transferred at room temperature onto a 0.45 μM polyvinylidene difluoride (PVDF) membrane (Bio-Rad Laboratories, Inc.) at 50 mA for 1 h. The membrane was blocked after transfer with 5% skim milk in Tris-buffered saline, 0.1% Tween 20 (TBST) buffer for 1 h at room temperature. The membrane was later incubated with mouse anti-6×His antibody (at a 1:5000 ratio dilution) for 1 h at room temperature. The membrane was then washed four times with TBST buffer and incubated with HRP-conjugated AffiniPure goat anti-mouse IgG (at 1:5000 ratio dilution) as a secondary antibody to detect the expression of rVP1 protein using an ECL detection kit and X-ray films (Konica Minolta, Gurugram, Haryana, India) exposed to the membranes before development in a Kodak X-OMAT 2000 processor (Kodak, Ontario, Canada).

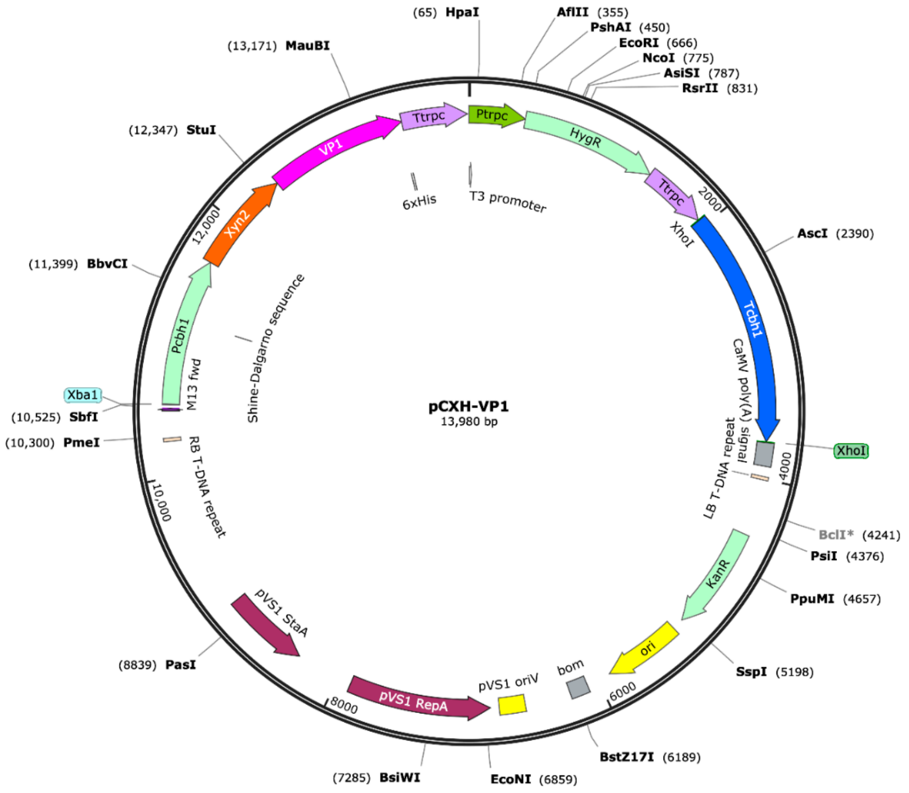

Supplementary Figure S1. Schematic representation of the constructed vector pCXH-Pcbh1-Xyn2-VP1-Ttrpc (pCXH-VP1). Pcbh1, *T. reesei cbh1* promoter; Xyn2, xynlanase 2 (*xyn2)* gene of *T. reesei*; VP1, *vp1* gene (w/o signal peptide sequence) of *G. lucidum*; TtrpC, *A. nidulans trpC* terminator. Fusing *vp1* with *xyn2* allowed for efficient extracellular secretion of rVP1 upon its endogenously mediated cleavage from the XYN2 protein.

Supplemental Table S1. Primers used for the construction of the pCXH-VP1 cloning vector.

Primer	Primer Sequence
PCBH1 F	5′GCCTGCAGGTCGACTCTAGATTTCCCTGATTCAGCGTACC3'
PCBH1 R	5′AGGGAGGTGAAGGAGACCATTTGACTATTGGGTTTCTGTG3′
XYN2 F	5′CACAGAAACCCAATAGTCAAATGGTCTCCTTCACCTCCCT3′
XYN2 R	5'AGAAGAGCTTTGAAGGCCATGCTGACGGTGATGGAAGCAG3'
VP1 F	5′CTGCTTCCATCACCGTCAGCATGGCCCTCACGCGCCGCGT3'
VP1 R	5′CGATCCGGTCGGCATCTACTTTAATGATGATGATGATGATGCACGGGGGCGACCGAGGTG3'
TTRPC F	5′CACCTCGGTCGCCCCCGTGCATCATCATCATCATCATTAAAGTAGATGCCGACCGGATCG3'
TTRPC R	5′AATTGCGCGGATCCTCTAGACAGGGCTGGTGACGGAATTT3'
TCBH1 F	5′CGTCACCAGCCCTGCTCGAGAGCTCCGTGGCGAAAGCCTG3'
TCBH1 R	5′TTATTATGGAGAAACTCGAGCTCGAGGCGTGTCTATTCAT3'

References

1. Blum, H.; Beier, H.; Gross, H.J. Improved silver staining of plant proteins, RNA and DNA in polyacrylamide gels. *Electrophoresis* **1987,** *8*, 93–99.
